# Sphingomyelin synthase 2 is a positive regulator of the CSF1R-STAT3 pathway in pancreatic cancer-associated macrophage

**DOI:** 10.3389/fphar.2022.902016

**Published:** 2022-10-17

**Authors:** Shuhua He, Xiang Gu, Jintong Yang, Fei Xu, Jiachun Hu, Wei Wang, Yiheng Huang, Bin Lou, Tingbo Ding, Lu Zhou, Deyong Ye, Ker Yu, Jibin Dong

**Affiliations:** ^1^ Department of Pharmacology and Biochemistry, School of Pharmacy, Fudan University, Shanghai, China; ^2^ Department of Medicinal Chemistry, School of Pharmacy, Fudan University, Shanghai, China; ^3^ Department of Clinical Medicine, Shanghai Jiaotong University of Medicine, Shanghai, China; ^4^ Experiment & Teaching Center, School of Pharmacy, Fudan University, Shanghai, China; ^5^ Shanghai Engineering Research Center of Immunotherapeutics, Fudan University, Shanghai, China

**Keywords:** sphingomyelin synthase 2, sphingomyelin synthase 2 inhibitor YE2, pancreatic ductal adenocarcinoma, tumor microenvironment, M2-like macrophages

## Abstract

**Background:** Tumor-associated macrophages (TAMs) are one of the most abundant immune cells in the pancreatic cancer stroma and are related to the poor prognosis of pancreatic ductal adenocarcinoma (PDAC) patients. Therefore, targeting tumor-associated macrophages is a possible strategy for the treatment of pancreatic cancer.

**Purpose:** We would like to investigate the role of sphingomyelin synthase 2 (SMS2) and the effect of the synthase 2 selective inhibitor YE2 in TAMs and the pancreatic tumor microenvironment. In addition, we also would like to investigate the mechanism by which YE2 attenuates macrophage M2 polarization.

**Methods:** YE2 was utilized to treat macrophages (*in vitro*) and mice (*in vivo*). Western blotting and real-time PCR were used to detect the protein levels and mRNA levels of macrophage M2 polarization markers and their downstream signaling pathways. Sphingomyelin synthase 2 gene knockout (KO) mice and their controls were used to establish a PANC-02 orthotopic pancreatic cancer model, and immune cell infiltration in the tumor tissue was analyzed by immunohistochemistry (IHC).

**Results:** We found that sphingomyelin synthase 2 mRNA expression is positively correlated with tumor-associated macrophages, the immunosuppressive microenvironment, and poor prognosis in pancreatic ductal adenocarcinoma patients. Sphingomyelin synthase 2 deficiency was confirmed to have an inhibitory effect on the growth of orthotopic PANC-02 tumors *in vivo*. The deficiency not only reduced the infiltration of tumor-associated macrophages but also regulated other immune components in the tumor microenvironment. In tissue culture, YE2 inhibited M2 polarization in both bone marrow-derived macrophages (BMDMs) and THP-1 macrophages and eliminated the protumor effect of M2 macrophages. In the mouse model, YE2 treatment reduced the infiltration of TAMs and regulated other immune components in the tumor microenvironment, slowing the progression of PANC-02 tumors. In terms of mechanism, we found that the inhibition of sphingomyelin synthase 2 could downregulate the expression of IL4Rα and CSF1R, thereby attenuating M2 polarization.

**Conclusion:** The sphingomyelin synthase 2 inhibitor YE2 or sphingomyelin synthase 2 deficiency can prevent macrophage M2 polarization in pancreatic cancer, and sphingomyelin synthase 2 could be a new potential target for the treatment of pancreatic cancer.

## Graphical Abstract

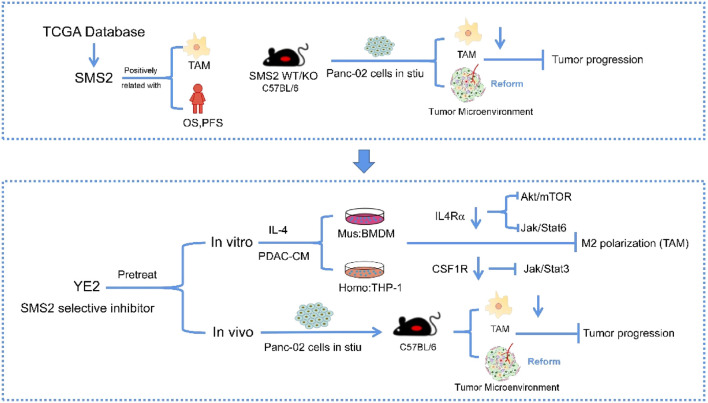

## Introduction

Pancreatic ductal adenocarcinoma (PDAC) is the most prevalent neoplastic disease in the pancreas, accounting for more than 90% of all pancreatic malignancies (Kleeff et al., 2016). To date, PDAC is the seventh leading cause of cancer-related deaths in the world, with a 5-year overall survival rate of less than 10% (Rawla et al., 2019; Siegel et al., 2020). Because of nonspecific symptoms and a lack of prognostic and diagnostic tumor markers, patients with PDAC are usually diagnosed at an advanced stage, making the disease worsen and incurable. Surgical resection is still the only treatment for PDAC; however, the proportion of patients with tumors that can be surgically removed is only approximately 10–20% (Aier et al., 2019).

PDAC has a highly immunosuppressive microenvironment characterized by a dense desmoplastic stroma. In some cases, the stromal tissue can account for 80% of the tumor. The components of the matrix include pancreatic stellate cells that produce collagen matrix (also called cancer-associated fibroblasts (CAFs)), infiltrating immune cells (e.g., bone marrow-derived suppressor cells, tumor-related macrophages, dendritic cells, Treg cells, *etc.*), endothelial cells and neuronal cells (Erkan et al., 2012). Due to the functional complexity of the PDAC microenvironment and the complex crosstalk between tumor cells and stromal cells, finding effective strategies to significantly and extensively regulate the pancreatic tumor microenvironment is crucial.

In the tumor stroma, tumor-associated macrophages (TAMs) are a type of macrophage that are more inclined to M2 polarization (Mantovani et al., 2002) or M2-like macrophages and play an important role in multiple stages and aspects of pancreatic cancer, such as tumor initiation, inflammation, immune evasion progression, metastasis, angiogenesis and chemotherapy resistance (Lankadasari et al., 2019). It has been reported that TAMs are one of the earliest infiltrating cells in pancreatic intraepithelial tumors (PAINs), and they increase persistently during the progression of aggressive cancer (Clark et al., 2007; Beatty et al., 2017). The preclinical PDAC mouse model has proven the importance of TAMs in driving angiogenesis, matrix remodeling, immunosuppression, tumor cell invasion and drug resistance (Mitchem et al., 2013; Sanford et al., 2013; Kalbasi et al., 2017; Zhu et al., 2017; Nywening et al., 2018). Importantly, in human PDAC patients, macrophage density is an independent prognostic factor and correlates with a higher risk of disease progression, recurrence, metastasis, and shorter overall survival (Yu et al., 2019). Therefore, TAMs could be a potential therapeutic target for pancreatic cancer treatment.

Sphingomyelin synthase 2 (SMS2), located on the plasma membrane, is the key enzyme for maintaining sphingomyelin (SM) levels there (Liu et al., 2009b; Yeang et al., 2011). Lipid rafts are important microdomain of cell membranes that provide a platform for many receptors and transport proteins. SM is an important component of plasma membrane lipid rafts (Kolesnick, 1991; Simons and Toomre, 2000; Zama et al., 2018). SMS2 plays an important role in the formation of lipid rafts. Under the stimulation of endotoxin and palmitate, SMS2 deficiency attenuated the activation of NF-κB and MAP kinases, indicating that SMS2 has a proinflammatory effect on macrophages (Hailemariam et al., 2008). Previously, we found that SMS2 deficiency or inhibition significantly attenuated macrophage M2 polarization and inhibited tumor growth and metastasis in a triple-negative breast cancer (TNBC) mouse model, reflecting the role of SMS2 in the M2 polarization of macrophages in breast cancer (Deng et al., 2021).

In this study, we examined the effects of YE2, as an SMS2 inhibitor, on TAMs *in vitro* and explored its mechanisms on macrophage M2 polarization. We also studied the effect of SMS2 deficiency and SMS2 inhibition on tumor growth and the immune microenvironment in a mouse model of PANC-02 orthotopic pancreatic cancer.

## Materials and methods

### Chemicals and inhibitors

The specific SMS2 inhibitor YE2 was designed and synthesized in the School of Pharmacy, Fudan University (CN202210213012.7). The activator of STAT3 is colivelin TFA (MCE, HY-P1061A).

### Bioinformatics analysis

The GEPIA website (http://gepia.cancer-pku.cn) was used to analyze the expression of SMS2 in normal samples from the GTEX database and pancreatic adenocarcinoma (PAAD) tumor samples from the TCGA database (Tang et al., 2017). PAAD TCGA data were downloaded *via* cBioPortal (http://www.cbioportal.org) and used to analyze the relationship between SMS2 expression and overall survival and progression-free survival, as well as the correlation between SMS2 expression and M2 biomarkers. The correlation between SMS2 and immune cell infiltration in the tumor microenvironment was analyzed by Timer2.0 (http://timer.comp-genomics.org) (Li et al., 2020).

### Cell lines and cell culture

PANC-1 and THP-1 cells were purchased from the Cell Bank of the Chinese Academy of Sciences, and luciferase-PANC-02 cells were purchased from Shanghai MEIXUAN Biological Science and Technology LTD. PANC-1 cells were grown in DMEM containing 10% FBS (Sigma), 1% glutamine and 1% sodium pyruvate. THP-1 cells were grown in RPMI-1640 containing 10% FBS and 0.05 mM β-mercaptoethanol. PANC-02 cells were grown in DMEM containing 10% FBS. All cell lines were incubated at 37 °C in a 5% CO2 atmosphere with saturated humidity.

### Establishment of a syngeneic orthotopic mouse model of pancreatic cancer

All animal studies were conducted under the approval of the IACUC of the School of Pharmacy of Fudan University. The SMS2 KO mice were a gift from Prof. Xian-Cheng Jiang (Liu et al., 2009a). The genotype of the SMS2 WT/KO mouse was identified by PCR. The specific primers for WT: forward, 5′-GTG​GCG​GAC​AAT​GGA​TAT​CAT​AGA​GAC​AGC-3′ and reverse, 5′-GATAAGG TCT​TGG​GTT​TGC​CCT​TGC​C-3'; for KO: forward, 5′-GCC​AGA​GGC​CAC​TTG​TGT​AGC-3′ and reverse, 5′-GAT​AAG​GTC​TTG​GGT​TTG​CCC​TTG​C-3'. Then, the SMS2 WT and SMS2 KO mice (5 mice in each group) were orthotopically implanted with C57BL/6 derived PANC-02 cells.

Six-week-old female C57BL/6 mice were purchased from SLRC Laboratory (Shanghai, China). The mice were randomly divided into two groups (5 mice in each group) and orally administered YE2 (10 mg/kg) or vehicle (0.5% sodium carboxymethyl cellulose) twice a day. Plasma SM was measured by a commercial kit (mlbio, SU-B20566). Two weeks after YE2 administration, we established an orthotopic tumor model as follows.

Avertin (Nanjing AIBI Bio-Technology Co., Ltd., 2023A) was used to anesthetize the mice. Then, the abdominal cavity was opened by 1.5-cm-wide lateral laparotomy pointing slightly to the right to identify the tail of the pancreas, and the tail was lifted up with a cotton swab. PANC-02 cells (1×10^6^), suspended in 50 μL PBS and Matrigel (Corning, 3,54,248) (1:1) solution, were slowly injected into the tail of the pancreas with an insulin syringe. After injection, the pancreas was returned to the abdominal cavity, and the wound was sutured. All mice were sacrificed on the 28th day, and the tumor tissues were harvested by dissection.

### Isolation of monocytes and differentiation of bone marrow-derived macrophages

C57BL/6 mice (6–8 weeks old) were euthanized *via* cervical dislocation and soaked in 75% ethanol for 2 min, and their tibia and femur were dissected. The muscle was aseptically removed, and then the bone cavity was flushed with a 25 G needle soaked with dulbecco’s modified eagle medium (DMEM) until it turned white. The bone marrow cells were collected and cultured in DMEM containing 30% L929 conditioned medium (L929 CM) and 10% FBS for 7 days. The complete medium was changed every 3 days, and the phenotype of mature macrophages was analyzed by flow cytometry.

### Differentiation of THP-1

THP-1 cells were cultured in RPMI-1640 mixed with 100 ng/ml PMA (absin, abs9107) and 10% FBS for 48 h and then replaced with complete medium without PMA for 24 h to differentiate into mature macrophages.

### Sphingomyelin synthase 2 enzyme activity determination

Macrophages were pretreated with 0, 0.33, 1.1, 3.3, 11, 33, 100 μM YE2. After 2 h, 2 μg/ml C6-NBD-ceramide (Sigma–Aldrich) was added. The supernatant was collected, and a solution of chloroform:methanol (volume ratio 2:1) was added for lipid extraction 2 h later. Then, the solution was centrifuged at 10,000 rpm for 10 min, and the lower organic phase was collected, dried with N_2_, and reconstituted in 30 μL chloroform. Finally, the product NBD-SM was separated by TLC using CHCL3:CH3OH:NH3·H2O, 16:4:1 (v/v/v) as the developing solvent. The bands were observed through UV light detection (Ding and Jiang, 2014).

### Preparation of conditioned medium

For the acquisition of the culture supernatant of L929 cells, cells were seeded with a confluency of 60%, and the culture supernatant was collected after 3 days. To obtain the conditioned medium of PDAC cells, PANC-02 and PNAC-1 cells were cultured to 90% confluence at first and then cultured with serum-free medium for 24 h. The supernatant was collected. For the conditioned medium of macrophages, the macrophages were cultured in DMEM or PDAC-CM with or without YE2 for 48 h. Then, the macrophages were washed twice with PBS and incubated in fresh DMEM without FBS for 24 h. The supernatant was then collected. All the supernatant collected was centrifuged at 5,000 rpm for 10 min and then filtered through a 0.22 μm filter membrane. The conditioned medium was stored at −80°C for later use.

### RNA isolation and RT–PCR

Total RNA was extracted and purified with an RNA isolator (Vazyme, R401-01) according to the standard protocol. Hifair II 1ST Strand cDNA Synthesis SuperMix (Yeasen, 11123ES60) was used to perform reverse transcription. Hieff UNICON qPCR SYBR Green Master Mix (NO Rox) (Yeasen, 11198ES08) was used to perform real-time quantitative PCR. The relative mRNA level of specific genes was normalized to the internal reference level. The primers used are listed in Table 1.

**TABLE 1:** The primers used in RT-PCR.

Organism	Target genes	Forward primer (5′-3′)	Reverse primer (5′-3′)
Mouse	CD206	CTC​TGT​TCA​GCT​ATT​GGA​CGC	GGAATTTC TGGGATTCAGCTTC
	Arg-1	TCC​CGC​TCT​AAG​CAC​ACC​A	CCG​GTT​CCG​TTT​GCT​CTC​C
	TGF-β	AGA​CCA​CAT​CAG​CAT​TGA​GTG	GGT​GGC​AAC​GAA​TGT​AGC​TGT
	IL4Rα	ACA​CTA​CAG​GCT​GAT​GTT​CTT​CG	TGG​ACC​GGC​CTA​TTC​ATT​TCC
	18S	GGC​CGT​TCT​TAG​TTG​GTG​GAG​CG	GGC​CGT​TCT​TAG​TTG​GTG​GAG​CG
Human	CD206	AGC​CAA​CAC​CAG​CTC​CTC​AAG​A	CAA​AAC​GCT​CGC​GCA​TTG​TCC​A
	CCL22	GAG​CAT​GGA​TCG​CCT​ACA​G	CAG​ACG​GTA​ACG​GAC​GTA​ATC
	FN-1	ACT​GTA​CAT​GCT​TCG​GTC​AG	AGT​CTC​TGA​ATC​CTG​GCA​TTG
	IL4Rα	GCA​GTG​GCA​TTG​TCT​ACT​CAG​C	GGTTGTAGGGGGCGAGGA
	GAPDH	GGG​CTG​CTT​TTA​ACT​CTG​GT	TGA​TTT​TGG​AGG​GAT​CTC​GC

### Western blotting

BMDMs and THP-1 cells were treated with IL-4 (MUS: Pepro Tech, 214–14; HUM: Pepro Tech, 200–04) or a 1:1 solution of PDAC-CM and complete medium containing 10% FBS for 48 h. NuPAGE™ LDS Sample Buffer (Invitrogen, NP0007) was directly added to the cell culture plate to lyse the cells on ice. The protein was heated in an iron bath at 70°C for 10 min, separated by sodium dodecyl sulfate–polyacrylamide gel electrophoresis (SDS–PAGE), and electrotransferred to a polyvinylidene fluoride (PVDF) membrane (Merck Millipore). The main antibodies used in the research were CD206 (ab64693, Abcam), Arg-1 (93,668, CST), p-Akt (4060S, CST), Akt (4691S, CST), p-Stat6 (ab263947, Abcam), Stat6 (ab32520, Abcam), p-mTOR (5,536, CST), mTOR (2,983, CST), mouse IL4Rα (MAB530, R&D system), human IL4Rα (MAB230, R&D system), CSF1R (ab254357, Abcam), p-Stat3 (ab76315, Abcam), and Ym-1 (ab192029, Abcam). The protein bands were observed using an enhanced chemiluminescence (ECL) detection kit (P10100, NCM), and images were obtained using a chemiluminescence detection system (Bio–Rad).

### Flow cytometry analysis

The cells were first incubated with F4/80 antibody, fixed with fixative (BioLegend, 420,801), washed twice with intracellular staining perm wash buffer (BioLegend, 421,002), and then incubated with anti-mouse CD206 to detect M2 polarized macrophages. The antibody was incubated at 4°C in the dark for 30 min. The antibodies for cell staining were PE-cyanine7 mouse anti-F4/80 (eBioscience, 25–4801-82) and APC mouse anti-CD206 (eBioscience; 17–2069-42).

### Immunohistochemistry

The tumor tissue was excised for paraffin embedding and sectioning (RIBIOLOGY, China). IHC was used to analyze the expression of some proteins. The antibodies used were F4/80 (Abcam, ab16911), CD206 (Abcam, ab64693), α-SMA (CST, 19,245), CD31 (Abcam, ab9498), FOXP3 (Abcam, ab215206), CD4 (Abcam, ab183685), CD8 (Abcam, ab217344), CD86 (Abcam, ab119857), and Granzyme B (Abcam, ab255598).

### *In vitro* SM treatment

SM (Sigma, 85,615) was dissolved in 2:1 ethanol-Me2SO to make a 5 mM stock. An aliquot of this stock solution was added to the culture medium to a final concentration of 40 μM as described previously (Puri et al., 2003).

### Pharmacokinetic studies

Compound YE2 was formulated for intravenous (10 mg/kg) in DMSO/castor oil/0.9% NaCl = 5/5/90 and oral administrations (20 mg/kg) in 0.5% carboxymethylcellulose sodium, which were subjected to pharmacokinetic studies on C57BL/6 mice. Serial specimens were collected 5, 15, and 30 min and 0.25, 0.5, 1, 2, 4, 6, and 8 h following intravenous and oral administration, respectively. The plasma concentration was quantified by LC–MS/MS. Pharmacokinetic parameters were calculated from the mean serum concentration by WinNonlin Professional Edition Version 2.1 (Mo et al., 2018).

### Statistical analysis

Except for the *in vivo* data presented as the mean ± standard error, all other data are presented as the mean ± standard deviation. Prism 6 (GraphPad Software, San Diego, CA, United States) was used for statistical analysis, and the two-tailed Student’s t test was used to analyze the significant differences between two groups. A *p* value < 0.05 was considered statistically significant; *p < 0.05; **p < 0.01; * * *p < 0.001; * * * *p < 0.0001.

## Results

### High expression of SMS2 was associated with abundant tumor-associated macrophages and poor prognosis in patients with pancreatic cancer

**FIGURE 1:**
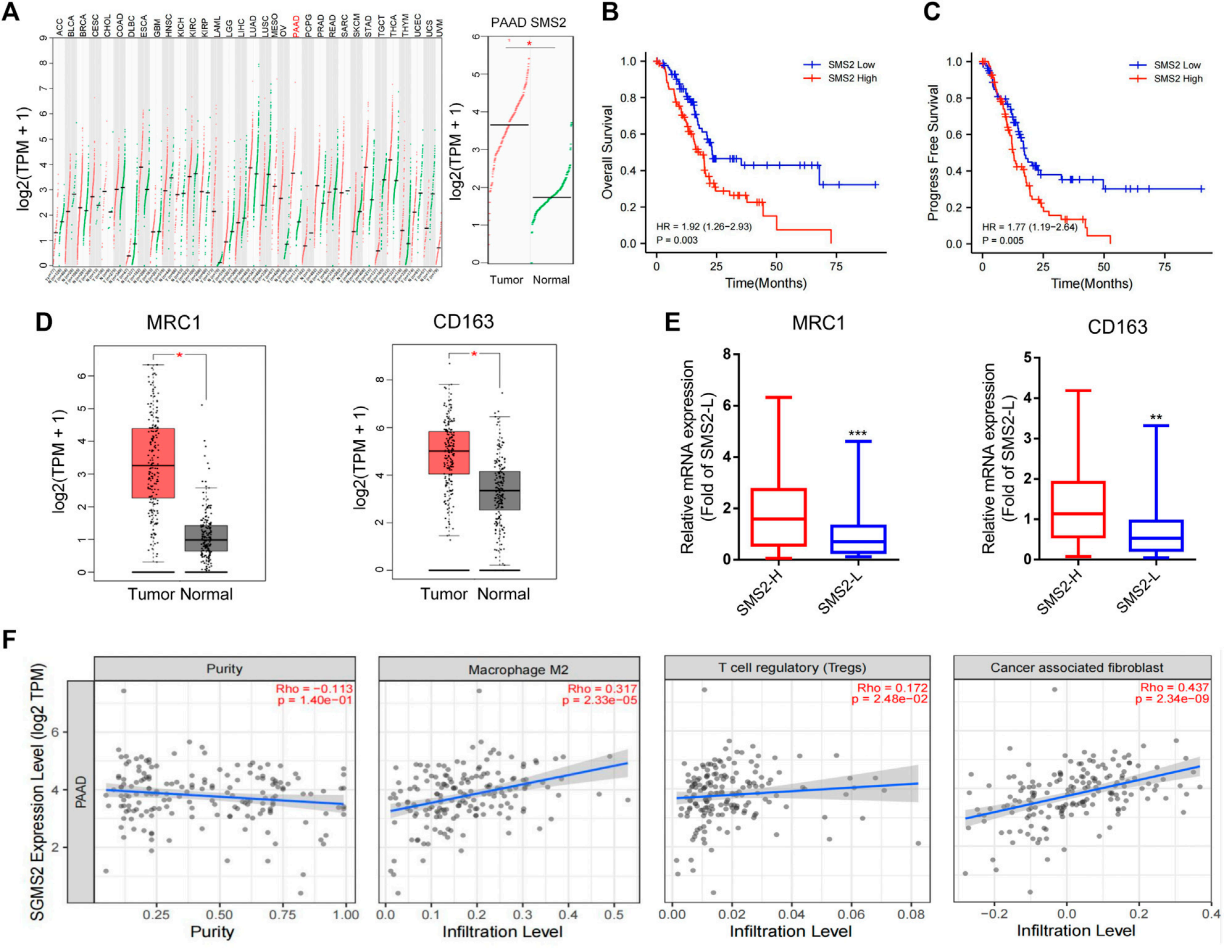
The high expression of SMS2 is related to the poor prognosis of pancreatic cancer patients.**(A)**. Based on data obtained from GEPIA, SMS2 is highly expressed in pancreatic cancer patients. The data of pancreatic cancer samples comes from the TCGA database. The normal samples come from the GTEX database. **(B,C and E)**. A total of 178 TCGA samples are divided into SMS2 High and SMS2 Low according to the median value of SMS2 expression **(B)** High expression of SMS2 is associated with lower overall survival rate, and **(C)** shorter progression-free survival. **(D)**. M2 polarization biomarkers MRC1 and CD163 are highly expressed in pancreatic cancer patients. **(E)**. High expression of SMS2 positively correlates with the expression of MRC1 and CD163. **(F)**. The expression of SMS2 positively correlates with the infiltration of M2 macrophages, Treg, CAF in pancreatic cancer. *p < 0.05; **p < 0.01; ***p < 0.001.

Through the GEPIA website, we found that SMS2 expression was significantly higher in the tumor tissue of pancreatic cancer patients than in normal human pancreatic tissue (Figure 1A). Utilizing the TCGA database, we also found that the overall survival rate (OS) (Figure 1B) and progression-free survival (PFS) (Figure 1C) of patients with high SMS2 expression (SMS2 High) were significantly lower than those of patients with low SMS2 expression (SMS2 Low), implying that SMS2 expression is related to the poor prognosis of pancreatic cancer. In addition, the expression of the TAM-related biomarkers MRC1/CD206 and CD163 was higher than that in normal human pancreatic tissue, indicating that TAMs are highly infiltrative in pancreatic cancer (Figure 1D). It was also discovered that the expression of TAM-related biomarkers in the SMS2 High group was significantly higher than that in the SMS2 Low group and the M2 macrophage fraction was higher in SMS2 High group (Supplementary Figure S1B), indicating that SMS2 expression is positively correlated with M2 polarization (Figure 1E). In addition, by using TIMER 2.0, we found that SMS2 expression was positively correlated with the expression of TAMs, Tregs, and CAFs in pancreatic tumors (Figure 1F), suggesting that SMS2 expression may play an important role in the microenvironment of pancreatic cancer.

### SMS2 deficiency prevented the growth of pancreatic carcinoma *in situ* by affecting the immune microenvironment

**FIGURE 2:**
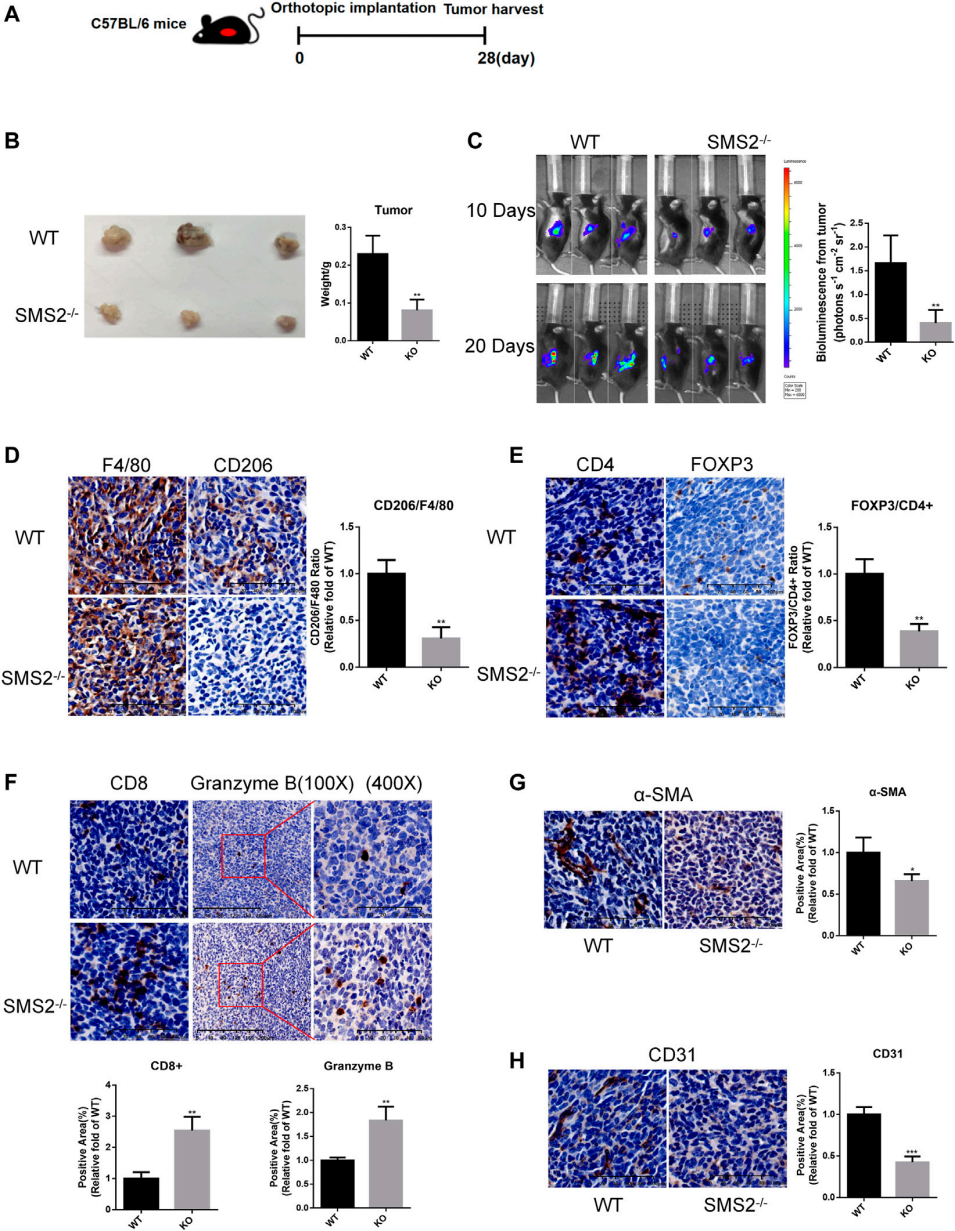
SMS2 knockout can inhibit the growth of PANC-02 orthotopic pancreatic tumor *in vivo*. **(A)**. Construction of orthotopic pancreatic tumor model of gene knockout mice. **(B)**. Tumor tissue harvested after 28 days. **(C)**. Representative bioluminescence images at 10th day and 20th day after tumor inoculation and quantitative analysis at 20th day. **(D)**. IHC representative images of M2 macrophages in tumor tissues and quantification. IHC representative images and quantification of immune cells CD4, FOXP3/CD4 **(E)**, CD8, Granzyme B **(F)**, CAF (α-SMA) **(G)** and vascular infiltration (CD31) **(H)** in tumor tissues. The results are shown as the mean ± SD. *p < 0.05; **p < 0.01; ***p < 0.001 (n = 5 mice/group). The observed field is 200X zoom of original IHC photo.

Based on the results of the database analysis above, we chose SMS2 gene knockout (KO) and WT mice to establish an orthotopic pancreatic cancer model to confirm that SMS2 deficiency plays an important role in PDAC progression. First, the genotype of SMS2 KO/WT mice was confirmed (Supplementary Figure S2F). After 28 days of implantation, the tumors of SMS2 KO mice were significantly smaller than those of WT mice (Figure 2B). The images of bioluminescence imaging at the 10th day and 20th day showed that SMS2 KO could delay the progression of pancreatic cancer (Figure 2C).

The tumor tissues were sectioned, and various immune components were stained and analyzed. The proportion of CD206+/F4/80 + TAM infiltration in the tumor tissues was significantly reduced (Figure 2D). For other immunosuppressive components, we found that the content of CD4+/Fopx3+ Treg cells was decreased (Figure 2E). And the infiltration of antitumor immune component CD8^+^ T cells was increased. Granzyme B secreted by CD8^+^ T cells and NK cells was also increased (Figure 2F). As CAFs play a role in promoting fibrosis in the process of pancreatic cancer, the infiltration of its marker α-SMA was reduced (Figure 2G). The content of CD31, a marker of vascular endothelial cells, was also decreased significantly (Figure 2H). These phenotypes confirmed that SMS2 deficiency plays an important role in TAM infiltration and the microenvironment of pancreatic cancer.

### Sphingomyelin synthase 2 inhibitor attenuated IL-4-induced macrophage M2 polarization

**FIGURE 3:**
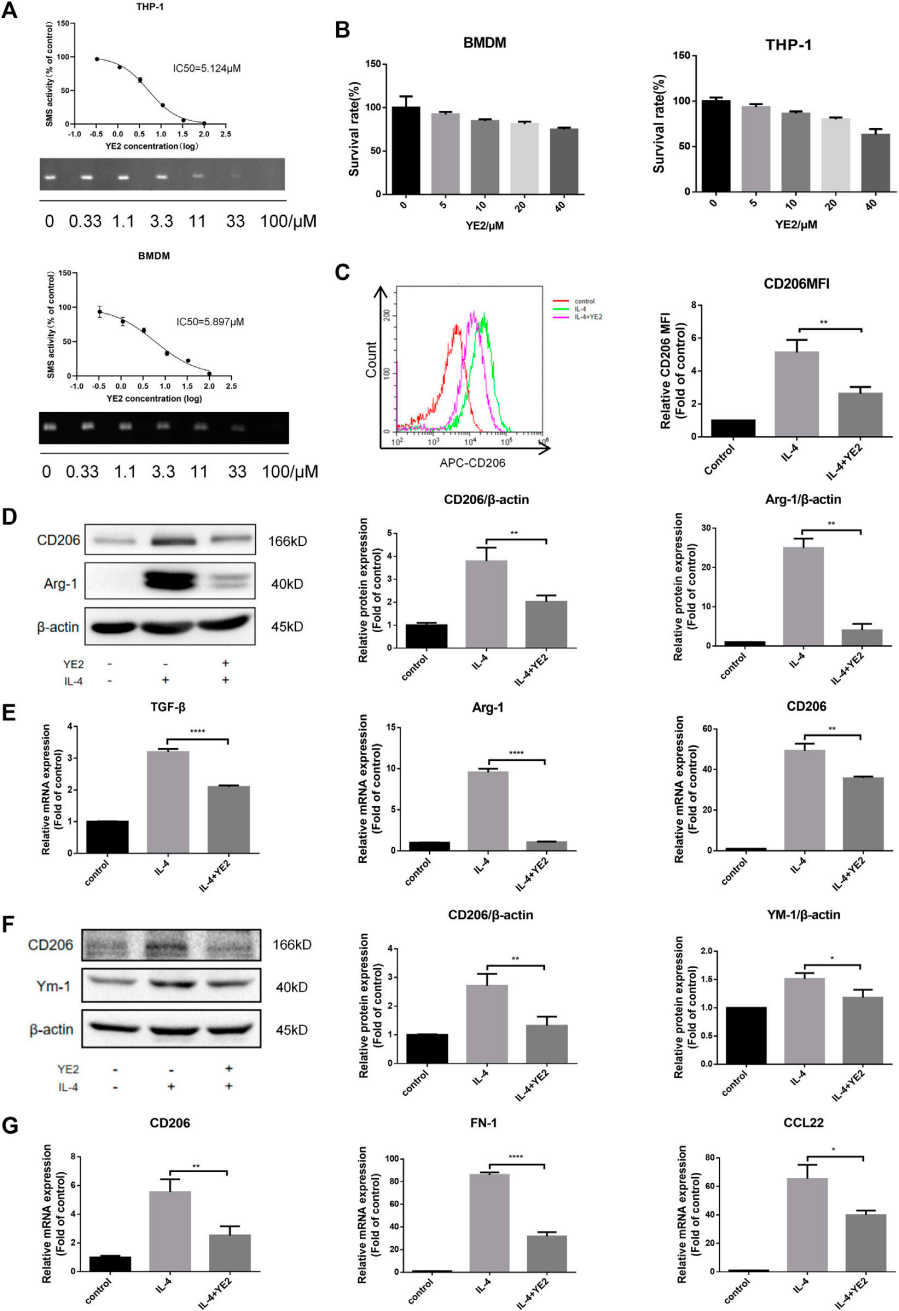
SMS2 selective inhibitor inhibits IL-4 induced M2 polarization *in vitro*. **(A)**. TLC measurements of SMS2 enzyme activity inhibition by YE2 in BMDM and THP-1. **(B)**. CCK8 measurements of the cell viability of BMDM and THP-1 with different concentration of YE2. **(C)**. Flow cytometric detection of M2 polarization induced by IL-4 for 48 h in BMDM with or without YE2 and quantification of CD206 mean fluorescence intensity (MFI) in BMDM. Immunoblotting of BMDM **(D)** and THP-1 **(F)** 48 h after IL-4 stimulation. Real-time PCR was performed 24 h after IL-4-stimulated M2 polarization in BMDM **(E)** and THP-1 **(G)**. Data is presented as the mean ± SD of three representative experiments. *p < 0.05; **p < 0.01; ***p < 0.001.

Next, we sought to investigate the consequences of SMS2 pharmacological inhibition employing an SMS2 inhibitor, YE2 (CN202210213012.7). Its inhibition selectivity on SMS2 were 800-fold higher than on its isozyme SMS1 (Supplementary Figure S3A). To obtain a more comprehensive verification, we chose human-derived macrophages, THP-1, and mouse bone marrow-derived macrophages, BMDMs, for the experiments. First, we found that YE2 exhibited a dose-dependent inhibitory effect on SMS2 activity, with IC50 values of 5.124 μM in THP-1 cells and 5.897 μM in BMDMs (Figure 3A) and we also verified its selectivity on SMS2 (Supplementary Figures S1H–J). We chose a concentration of 15 μM, which is 3 times the IC50, and the survival rate was over 80% for the following experiments (Figure 3B). But the cytotoxic effect of YE2 was much less when macrophage was treated with PDAC-CM meanwhile, which is consistent with subsequent experimental condition (Supplementary Figure S1D). After 2 h of drug pretreatment, IL-4 was added to induce M2 polarization. We found that in BMDMs, YE2 treatment significantly reduced CD206 MFI in BMDMs compared with the control (Figure 3C). We also measured M2 polarization markers and found that CD206 and Arg-1 at the protein level (Figure 3D) and CD206, Arg-1, and TGF-β at the mRNA level were significantly reduced (Figure 3E) compared with those in the control. In THP-1 cells, the M2 polarization markers CD206 and YM-1 at the protein level (Figure 3F) and CD206, CCL22, and FN-1 at the mRNA level were also significantly lower than those in control cells (Figure 3G). These results indicated that the SMS2 inhibitor YE2 attenuated the M2 polarization stimulated by IL-4.

### Sphingomyelin synthase 2 inhibitor attenuated macrophage M2 polarization induced by tumor-conditioned medium

**FIGURE 4:**
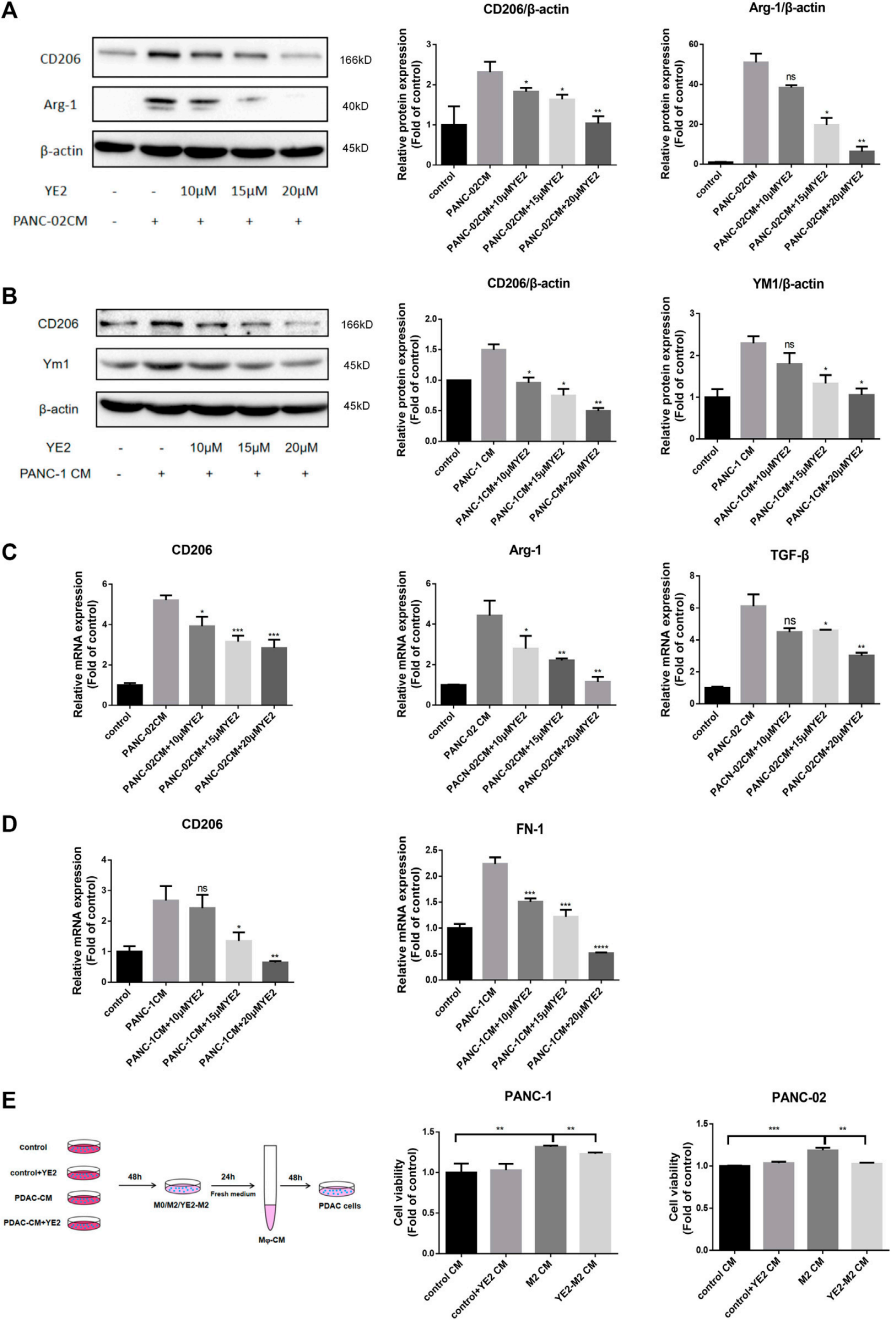
SMS2 inhibitor attenuates M2 polarization induced by CM of pancreatic cancer cells cultured *in vitro*. The conditioned medium of PANC-02 and PANC-1 was used to culture BMDM **(A)** and THP-1 **(B)** with or without YE2, respectively, and the cells were analyzed by immunoblotting after 48 h. The M2 polarization of BMDM **(C)** and THP-1 **(D)** was detected by Realtime PCR after 24 h. **(E)**. Conditioned medium of THP-1 and BMDM incubated in PDAC-CM with or without YE2 was collected to culture PANC-1 and PANC-02 cells. Data is presented as the mean ± SD of three representative experiments. *p < 0.05; **p < 0.01; ***p < 0.001; ****p < 0.0001.

To mimic the tumor microenvironment, we cultured BMDMs and THP-1 cells with conditioned medium from mouse-derived PANC-02 (PANC-02-CM) and human-derived PANC-1 (PDAC-CM) cells. In BMDMs, PANC-02-CM induced an increase in the expression of the M2 polarization markers CD206 and Arg-1 at the protein level (Figure 4A), and CD206, Arg-1, and TGF-β at the mRNA level (Figure 4C). After treatment with 10, 15, 20 μM YE2, these M2 polarization markers were reduced in a dose-dependent manner (Figures 4A,C). We also observed similar observations in THP-1 cells (Figures 4B,D) when PANC-1-CM was used. Furthermore, we found that the conditioned medium of M2 macrophages induced by PDAC-CM promoted PANC-1 and PANC-02 cell growth, and the conditioned medium of M2 macrophages incubated with 15 μM YE2 did not affect cell growth (Figure 4E). All these observations indicated that the SMS2 inhibitor can attenuate PDAC-CM-induced macrophage M2 polarization thereby diminishing its tumor-promoting effect.

### Sphingomyelin synthase 2 inhibitor prevented orthotopic pancreatic carcinoma growth and regulated its immunomicroenvironment

**FIGURE 5:**
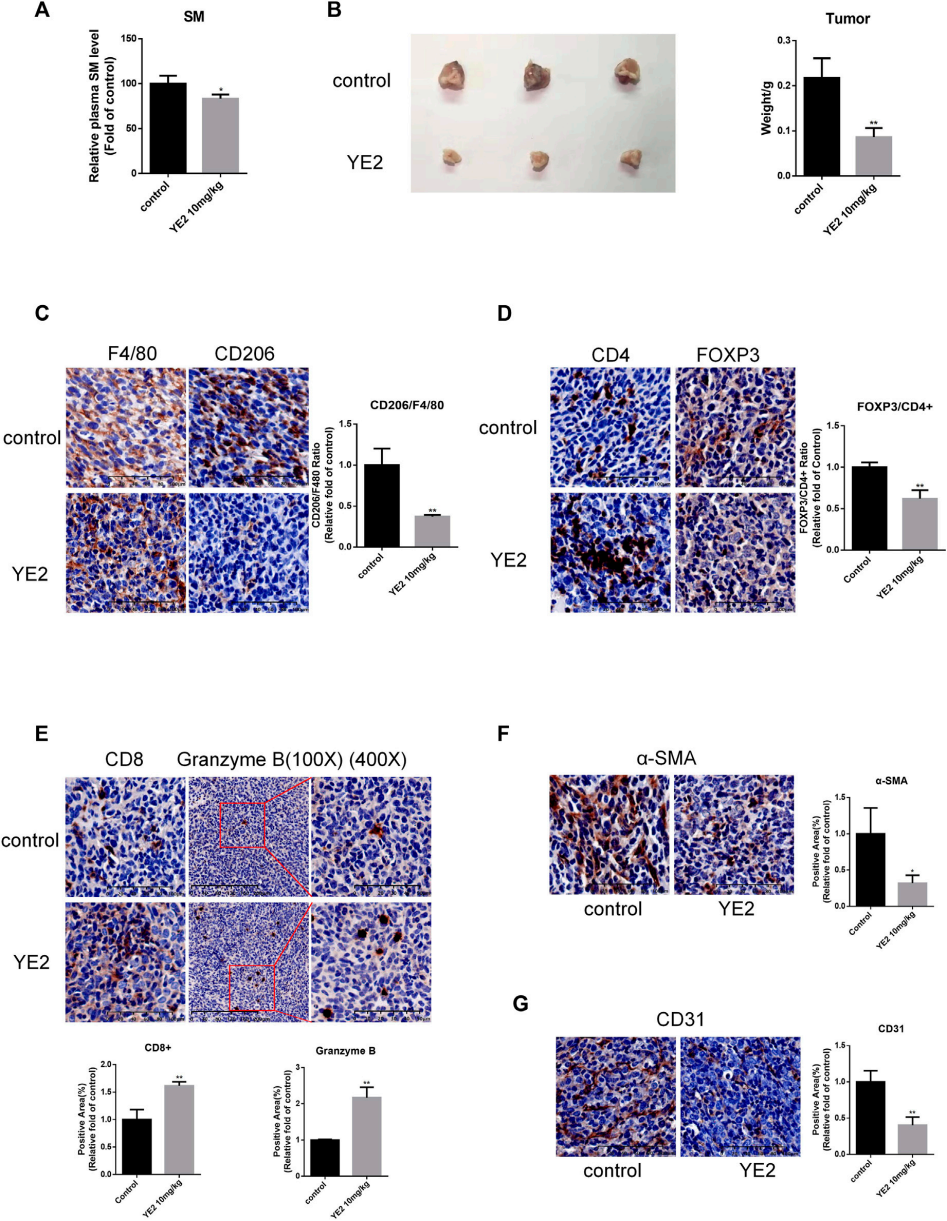
SMS2 inhibitor inhibits the growth of PANC-02 pancreatic orthotopic tumors *in vivo*. **(A)**. After pre-administration of SMS2 inhibitor at 10 mg/kg for 2 weeks, blood was taken, and the plasma SM level was measured with the SM ELISA kit. **(B)**. Tumor tissue harvested after 28 days. **(C)**. IHC analysis of M2 macrophage infiltration in tumor tissues of control group and YE2 group. IHC representative graph and quantifications of immune cells and components CD4, FOXP3/CD4 **(D)**, CD8, Granzyme B **(E)**, CAF (α-SMA) **(F)** and vascular (CD31) **(G)** infiltration in tumor tissues. The results are shown as the mean ± SD. *p < 0.05; **p < 0.01. (n = 5 mice/group). The observed field is 200X zoom of original IHC photo.

Next, we sought to investigate the *in vivo* effect of the SMS2 inhibitor. Before we started the experiment with YE2, we confirmed that it had no cytotoxicity on PANC-1 and PANC-02 cells (Supplementary Figure S1G). The pharmacokinetic test results of YE2 are shown in Supplementary Figure S2G. The drug dose was pre-determined to be 10 mg/kg based on a previous study and the results of pharmacokinetic and pharmacodynamic experiments. Our experiment was designed as shown in Supplementary Figure S2H. We measured plasma SM levels to monitor the efficacy of the inhibitor (Figure 5A). 2 weeks after drug treatment, PANC-02 cells were orthotopically implanted. We sacrificed the mice at Day 42 and found that the tumors were significantly smaller with the administration of YE2 (Figure 5B).

We also evaluated the TAM infiltration ratio, which was significantly reduced in the YE2 group (Figure 5C). Moreover, the content of immunosuppressive CD4+/Fopx3+ Treg cells was decreased (Figure 5D). The content of antitumor immune components, such as CD8^+^ T cells, was increased. Granzyme B was also increased (Figure 5E). The content of α-SMA was decreased (Figure 5F), and CD31 was also decreased significantly (Figure 5G). All these results indicated that the SMS2 inhibitor prevented the infiltration of TAMs and changed the composition of the tumor microenvironment, thus slowing the growth of PANC-02 tumors.

### Sphingomyelin synthase 2 inhibitor attenuated macrophage M2 polarization by influenceing CSF1R-STAT3 pathway

**FIGURE 6:**
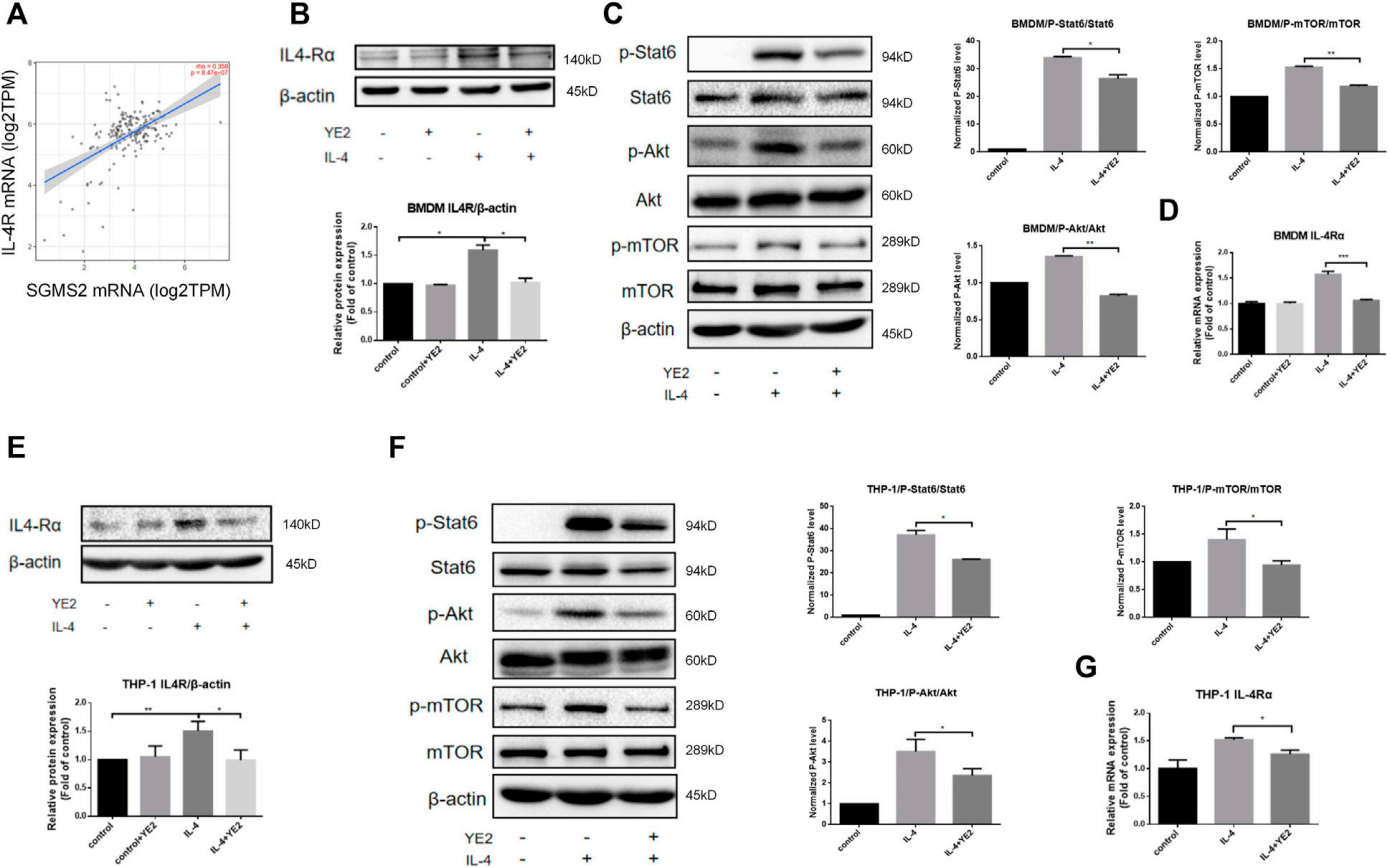
The mechanism of SMS2 inhibitors in inhibiting IL-4-induced M2 polarization. **(A)**. The expression of IL-4Rα is positively correlated with SMS2 in pancreatic cancer. Immunoblotting was used to detect the expression of IL-4Rαand downstream proteins in BMDM **(B and C)** and THP-1 **(E and F)** with or without YE2 48 h after IL-4 stimulation. The densitometry analysis data of BMDM **(B and C)** and THP-1 **(E and F)** was showed. Realtime PCR was used to detect the mRNA expression of IL-4Rα in BMDMs **(D)** and THP-1 **(G)** 24 h after IL-4 stimulation. The results are shown as the mean ± SD. *p < 0.05; **p < 0.01.

Then, we further explored the mechanism of the SMS2 inhibitor YE2 in attenuating M2 polarization induced by IL-4 or PDAC-CM. Based on Timer2.0 analysis, we learned that SMS2 is positively correlated with IL4Rα expression in pancreatic cancer (Figure 6A). We confirmed this finding in BMDMs and found that the protein level (Figure 6B) and mRNA level (Figure 6D) of IL4Rα were increased after IL-4 induction and reduced after subsequent YE2 treatment. (Figures 6B,D). Similar results were found in whole cell (Supplementary Figure S3C) and cell membrane (Supplementary Figure S3B) of SMS2 KO BMDMs. It is well known that the IL-4-mediated activation of the Akt/mTOR and Jak/Stat6 pathways is related to macrophage M2 polarization (Figure 6C) (Heller et al., 2008; Weisser et al., 2011; Covarrubias et al., 2016). We found that both were inhibited in YE2 treated BMDMs (Figure 6C) and THP-1 cells (Figures 6E–G). Similar phenotypes were also observed in SMS2 KO BMDMs (Supplementary Figure S3E).

**FIGURE 7:**
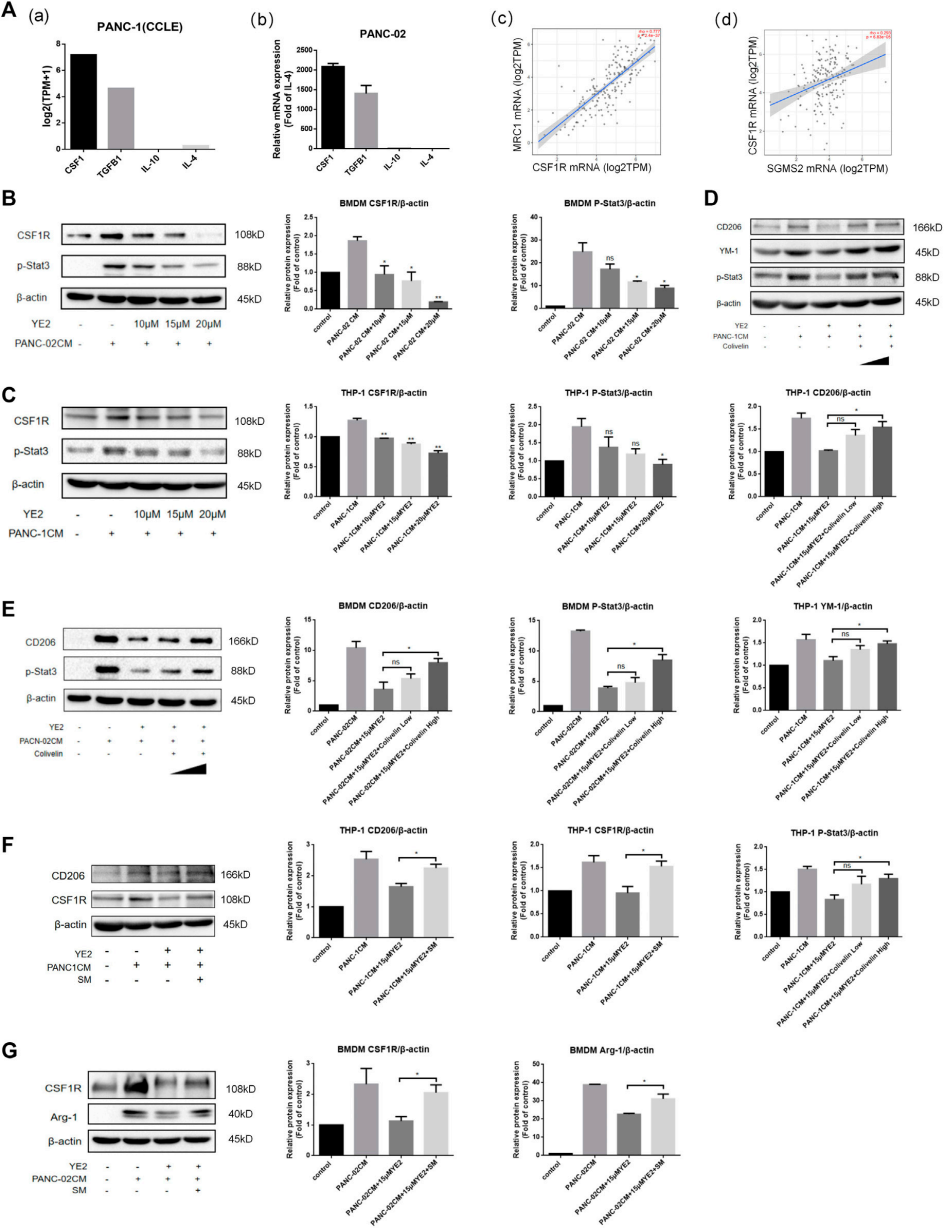
The mechanism of SMS2 inhibitors in inhibiting PANC-CM-induced M2 polarization. **(A)**. Gene expression of the cytokine that can induce M2 polarization in PANC-1 (a) was analyzed by CCLE database and was detected by Realtime PCR in PANC-02 (b). The expression of MRC1 is positively correlated with CSF1R (c). The expression of CSF1R is positively correlated with SMS2 in pancreatic cancer (d). Immunoblotting was used to detect the expression of CSF1R and downstream proteins in BMDM **(B)** and THP-1 **(C)** after PDAC-CM stimulation for 48 h with or without YE2. Immunoblotting was used to detect the expression of CD206 and p-Stat3 in BMDM **(E)** and THP-1 **(D)** after rescue of p-Stat3 with Colivelin. Immunoblotting was used to detect the expression of CSF1R and M2 marker (Arg-1 or CD206) after supplement of exogenous sphingomyelin (SM) in BMDM **(G)** and THP-1 **(F)**. The densitometry analysis data of BMDM **(B,E and G)** and THP-1 **(C,D and F)** was showed. *p < 0.05; **p < 0.01; ***p < 0.001.

In the study of macrophage M2 polarization induced by PDAC-CM, we found that CSF1 mRNA expression was higher than that of other cell cytokines related to M2 polarization in PANC-1 cells through CCLE database analysis [Figure 7A(a)] and the CSF1 secretion was also higher than other cytokines by ELISA (Supplementary Figure S1F). We also found that CSF1 mRNA expression was higher in PANC-02 cells by real-time PCR experiments [Figure 7A(b)]. Furthermore, Timer2.0 analysis also showed that the expression of CSF1R, the receptor of CSF1, mainly expressed on macrophages and monocytes, was positively related to the expression of the M2 polarization marker MRC1 in pancreatic cancer [Figure 7A(c)]. Furthermore, we found that SMS2 expression correlated positively with the expression of CSF1R [Figure 7A(d)]. Importantly, we found that PDAC-CM increased the expression of CSF1R in BMDMs (Figure 7B) and THP-1 cells (Figure 7C) and reduced it after subsequent treatment in a dose-dependent manner. SMS2 knock-out can also inhibit CSF1R expression induced by PDAC-CM in whole cell (Supplementary Figure S3C) and cell membrane. (Supplementary Figure S3B) of BMDMs. We examined the downstream proteins of CSF1R and found that the levels of p-Akt and p-Stat3 were increased in BMDMs with PDAC-CM induction and decreased in a dose-dependent manner with administration of YE2 (Figure 7B, Supplementary Figure S2A) (Mantovani et al., 2002; Hamilton, 2008; Huynh et al., 2012; Yuan et al., 2014; Vergadi et al., 2017). Similar results were confirmed in THP-1 cells, as shown in Figure 7C and Supplementary Figure S2B. Besides, we found SMS2 knock-out can inhibit p-Stat3 expression in BMDMs induced by PDAC-CM (Supplementary Figure S3D). To further confirm that the SMS2 inhibitor affects macrophage M2 polarization through the Jak/Stat3 pathway, we used colivelin at a low dose of 4 μM and a high dose of 8 μM to perform a rescue experiment of Stat3 on BMDMs (Figure 7E) and THP-1 cells (Figure 7D). The results showed that the level of p-Stat3 was restored, and M2 polarization markers were also recovered, suggesting that the SMS2 inhibitor attenuates CM-induced M2 polarization by inhibiting the Jak/Stat3 pathway. With supplementation of 40 μM exogenous SM, the protein levels of CSF1R and M2 markers (Arg-1 or CD206) were restored in BMDMs (Figure 7G) and THP-1 cells (Figure 7F), which proved that SM plays an important role in the expression of CSF1R.

## Discussion

SM is a key component of lipid rafts, which are closely related to the distribution of receptors and signal transduction on the membrane. In recent years, the excessive increase in SM content has been confirmed to be directly related to the occurrence, growth and immune evasion of cancer. The synthesis and hydrolysis of SM are involved in carcinogenesis and promotion of metastasis (D'Angelo et al., 2018). SMS2 is a key enzyme regulating membrane SM content and sphingomyelin metabolism. Our team had been working on SMS2, and more specifically the impact of SMS2 on lipid metabolism and atherosclerosis for more than 10 years. We have reported the impact of SMS2 on plaque stability, lipid infiltration, endothelial cell apoptosis, inflammatory pathways et al. (Hailemariam et al., 2008; Liu et al., 2009a; Liu et al., 2009b; Zhang et al., 2018). As a membrane protein, SMS2 may have impact on cellular lipid distribution, and immune cell function. Previous study has found that inhibition of SMS2 activity remodels the tumor microenvironment, slowing the progression and reducing metastasis of TNBC (Deng et al., 2021). PDAC is a cold tumor with a typical inhibitory tumor microenvironment and remodeling the immune cell infiltration may be an effective strategy for limiting the progress of PDAC. Hence, we tried to explore the possibility of influencing macrophage polarization and remodeling microenvironment with SMS2 regulation to treat PDAC.

The immune inhibitory composition of the PDAC tumor microenvironment, especially the high infiltration of TAMs, is an important factor in the immunosuppression and insensitivity of PDAC to various therapeutic drugs (Poh and Ernst, 2021). Through TCGA and GTEX database analysis, we found that TAMs were highly infiltrated in PDAC. SMS2 mRNA expression was also significantly higher in tumor tissue than in normal pancreatic tissue. In addition, patients with high SMS2 expression had higher TAM marker expression, suggesting that the high expression of SMS2 is related to the high infiltration of TAMs in pancreatic cancer patients. In addition, we found that the OS and PFS of patients with high SMS2 expression were lower than those with low expression, indicating that PDAC patients with high SMS2 expression have a poor prognosis. SMS1, an isozyme of SMS2, is mainly located in Golgi apparatus (Yeang et al., 2011). It would also alter the cellular SM level (Jin, et al., 2008; Li, et al., 2012) and its expression has strong correlation with TAM, Treg and CAF signatures as evaluated by deconvolution of RNA-seq data from tumor samples (Supplementary Figures S4C,D), similar to the effect of SMS2 expression. However, it did not affect the OS and PFS of patients with PDAC (Supplementary Figure S4B). We think the reason of this discrepancy is that two isozymes function differently and SMS1 may mediated more complex biological functions. It has been formerly found that SMS1 KO mice exhibited moderate neonatal lethality while SMS2 KO mice lives “normally” (Yano, et al., 2011; Li, et al., 2012). Besides, liver SMS1 deficiency results into GlcCer accumulation, stimulates β-catenin translocation into nuclei, and promotes tumorgenesis. While Liver SMS2 deficiency does not accumulate Glu-Cer (Li, et al., 2021). Additionally, there are some other reports indicating that down-regulation of SMS1 could promote tumor progression. (Brandan, et al., 2018; Bilal, et al., 2019). Based on the above analysis, we decided to focus on SMS2 and assumed that the specific inhibition of SMS2 may attenuate macrophage M2 polarization and regulate the immune microenvironment, thereby affecting pancreatic tumor growth.

In accord with our assumption, SMS2 deficiency led to smaller tumors and less TAM infiltration in mice. Previous studies have reported that mutual regulation exists among TAMs and other stromal cells, such as CAFs (Kuen et al., 2017; Zhang et al., 2017), and immune cells, such as T cells (Ruffell et al., 2014; Beatty et al., 2015; Daley et al., 2017), in the microenvironment of pancreatic cancer. It was indeed observed that with the decrease in M2 macrophage infiltration, the important immunosuppressive CD4+/FOXP3+ Treg cells were decreased, immune-activating CD8^+^ T cells were increased, granzyme B secreted by CD8^+^ T cells and NK cells was increased, and CAFs were decreased, indicating a remodeled microenvironment. In addition, some studies have found that TAMs are positively correlated with angiogenesis in PDAC (Dineen et al., 2008; Qian and Pollard, 2010). The vascular endothelial cells in the tumor were also decreased, indicating an antiangiogenic effect. Importantly, in this study, we used the new SMS2-specific small molecule inhibitor YE2 for the first time synthesized by Professor Zhou Lu’s research group from the School of Pharmacy, Fudan University. YE2 is a new SMS2 inhibitor compound optimized from Ly93 as the lead compound. Compared with Ly93, it has lower IC50 in purified enzyme level (Ly93 IC50: 0.091mM, YE2 IC50: 0.069 mM) and cell level (Ly93 IC50: 25mM, YE2 IC50: 5 mM). Besides, YE2 has better pharmacokinetic properties, such as improved bioavailability. (Li et al., 2019). By pretreatment of YE2, the M2 polarization induced by PDAC-CM or IL-4 in BMDMs and THP-1 cells was significantly inhibited, and the *in vivo* efficacy was consistent with that of SMS2 deficiency, suggesting the potential application of the SMS2-targeting small molecule inhibitor YE2 in pancreatic cancer treatment.

Then, to determine why SMS2 inhibition can attenuate macrophage M2 polarization, we studied the mechanism. It was found that YE2 can inhibit the up-regulation of IL4Rα induced by IL-4 on the membrane of macrophages, which leads to the down-regulation of the downstream Jak/Stat6 and Akt/mTOR signaling pathways related to M2 polarization. For the polarization induced by PDAC-CM, given the complex composition of cytokines secreted by tumor cells, we noticed that CSF1 plays a key role in inducing the M2 polarization of macrophages in PDAC (Zhu et al., 2014) and CSF1 mRNA was highly expressed in PANC-1 and PANC-02 cells. Actually, CSF1 can induce CSF1R expression in PDAC (Yeung et al., 2021). CSF1R belongs to receptor tyrosine kinase family (Stanley and Chitu, 2014). When the ligand binds to receptor tyrosine kinase, the receptor needs to dimerize on lipid rafts for activation (Casaletto and McClatchey, 2012). That is to say CSF1R depends on the presence of lipid rafts for activation. By maintaining SM level, SMS2 can affect the formation of lipid rafts where CSF1R is activated. In view of these facts, we speculate that down-regulating the activity of SMS2 may influence the activation of CSF1R through reducing distribution of CSF1R in lipid rafts, therefore attenuating the action of CSF1 and macrophage M2 polarization. Based on this speculation, we conducted the TCGA database analysis and learned that SMS2 is positively correlated with the expression of CSF1R in pancreatic cancer. The experimental results also proved that inhibition of SMS2 activity can downregulate CSF1R expression induced by PDAC-CM on macrophages, thus affecting the M2 polarization-related downstream PI3K/Akt and Jak/Stat3 signaling pathways. In addition, considering that SM synthesized by SMS2 participates in the formation of lipid rafts and affects signal transduction on the membrane, we observed that the expression of CSF1R and Arg-1 inhibited by YE2 was restored after exogenous SM addition. This finding indicates that the SM level may contribute to the expression of CSF1R. Through lipid raft extraction, we preliminarily found that PDAC-CM can stimulate the aggregation of CSF1R in rafts (Supplementary Figure S2D). But the treatment of SMS2 inhibitor reduced the content of CSF1R in rafts, which may be directly related to the reduction of SM content on the plasma membrane caused by YE2. The reduction of membrane SM limited the aggregation of CSF1R in rafts, and may also affect the stability of CSF1R in rafts. The increase of caveolin-1 still indicated that PDAC-CM treatment might have certain stimulation effect on rafts, although caveolin-1 cannot be used to quantify lipid rafts. All these results are consistent with our speculation, but the further specific research are still need to be done to find the mechanism behind them.

The limitation of current study is that we did not investigate the lipid composition alterations, and prove the selectivity through cellular lipidome analysis after SMS2 inhibition. Further LC-MS study should be performed to understand how YE2 regulates SM levels. In addition, our study also revealed that YE2 injection in mice with established tumors can reduce tumor growth (Supplementary Figure S5). Further investigations could be prepared to understand more comprehensively how SMS2 remodels the tumor microenvironment. To this end, we will use RNA-seq and single-cell sequencing technologies to study the impact of altered SMS2 activity on the immune cell composition of the pancreatic cancer microenvironment. As we also found a link between SMS2 and angiogenesis in our experiments, we will establish a pancreatic cancer metastasis model in the future to explore the effect of SMS2 activity inhibition on pancreatic cancer metastasis. Further exploration of the molecular mechanism by which SMS2 influences cell membrane composition and structure is needed.

## Conclusion

Overall, SMS2 deficiency can reduce the infiltration of TAMs in the pancreatic cancer microenvironment, as well as the infiltration of other immunosuppressive cells, such as Tregs and CAFs, thereby significantly slowing tumor progression. The new specific SMS2 small molecule inhibitor YE2 (CN202210213012.7) used in this study can attenuate macrophage M2 polarization by inhibiting the expression of IL4Rα and CSF1R on the plasma membrane and decreasing the infiltration of TAMs in pancreatic tumors, which changes the composition of the TME and suppresses tumor growth. Our findings provide a new potential target for antitumor immunotherapy of pancreatic cancer.

## Data Availability

The data analyzed in this study is subject to the following licenses/restrictions: This manuscript utilizes proprietary data. Requests to access these datasets should be directed to (Jibin Dong, jbdong@fudan.edu.cn).

## Abbreviations

PDAC, pancreatic ductal adenocarcinoma; TAM, tumor-associated macrophages; SMS2, sphingomyelin synthase 2; BMDM, bone marrow-derived macrophage; PanIN, pancreatic intraepithelial neoplasia; SM, sphingomyelin; TNBC, triple-negative breast cancer; PAAD, pancreatic adenocarcinoma; FBS, fetal bovine serum; PMA, phorbol ester; TLC, thin-layer chromatography; PBS, phosphate-buffered saline; CAF, cancer-associated fibroblasts; IHC, immunohistochemistry; DMEM, dulbecco’s modified eagle medium; ELISA, enzyme linked immunosorbent assay; PVDF, polyvinylidene fluoride; SDS-PAGE, sodium dodecyl sulfate–polyacrylamide gel electrophoresis; ECL, enhanced chemiluminescence; Arg-1, Arginase 1; CD206, mannose receptor; CD31, platelet endothelial cell adhesion molecule-1; CSF1, colony stimulating factor 1; FN-1, Fibronectin 1; YM1, Chitinase 3 like protein 3; α-SMA, α smooth muscle actin.
