# Supplementary figures and images for: Sphingomyelin synthase 2 is a positive regulator of the CSF1R-STAT3 pathway in pancreatic cancer-associated macrophage
Source: Front Pharmacol. 2022 Oct 17;13:902016. doi: 10.3389/fphar.2022.902016 (PMC9618885; doi:10.3389/fphar.2022.902016)

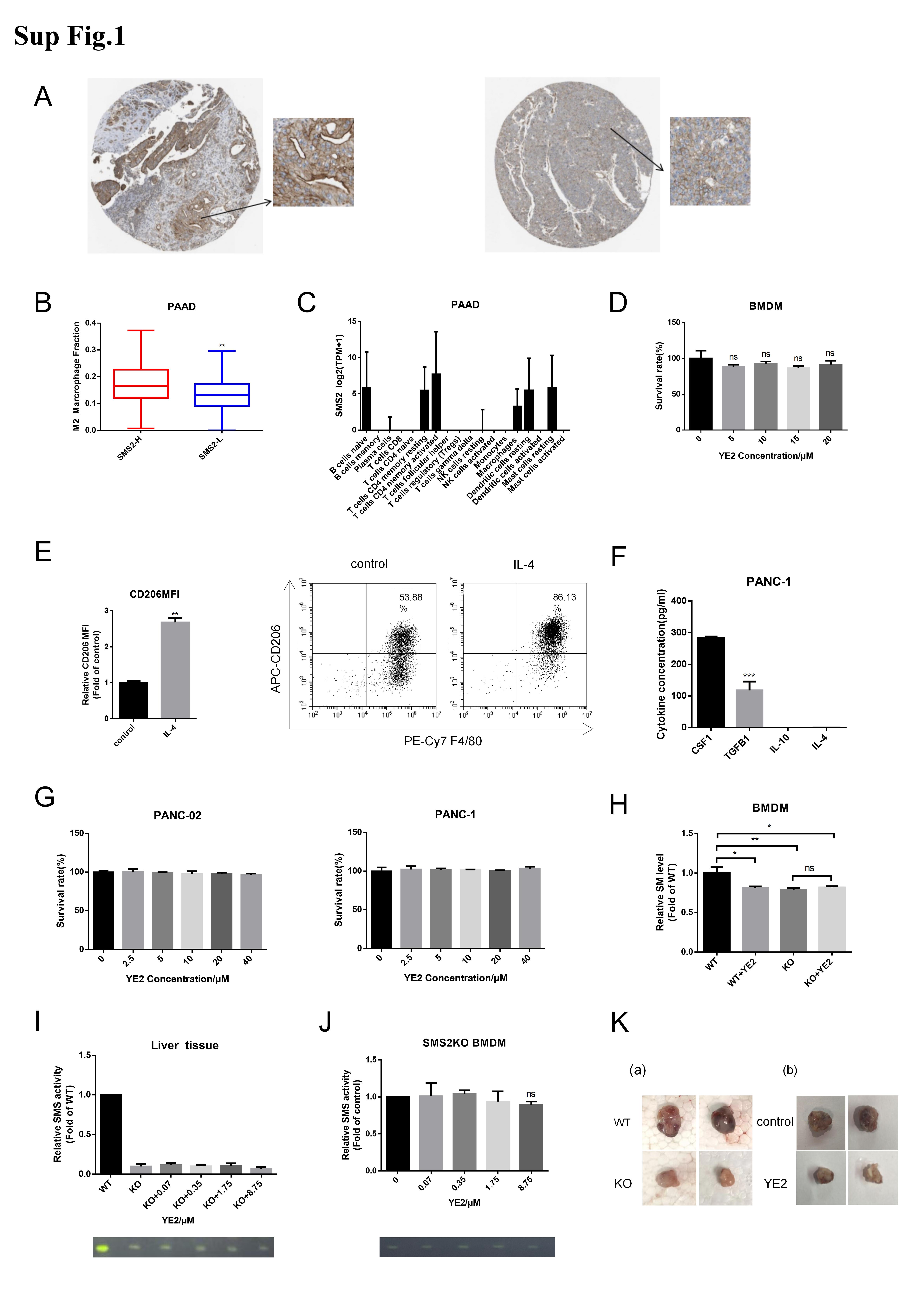

Supplement: Supplementary file 1 [file Image3.JPEG]

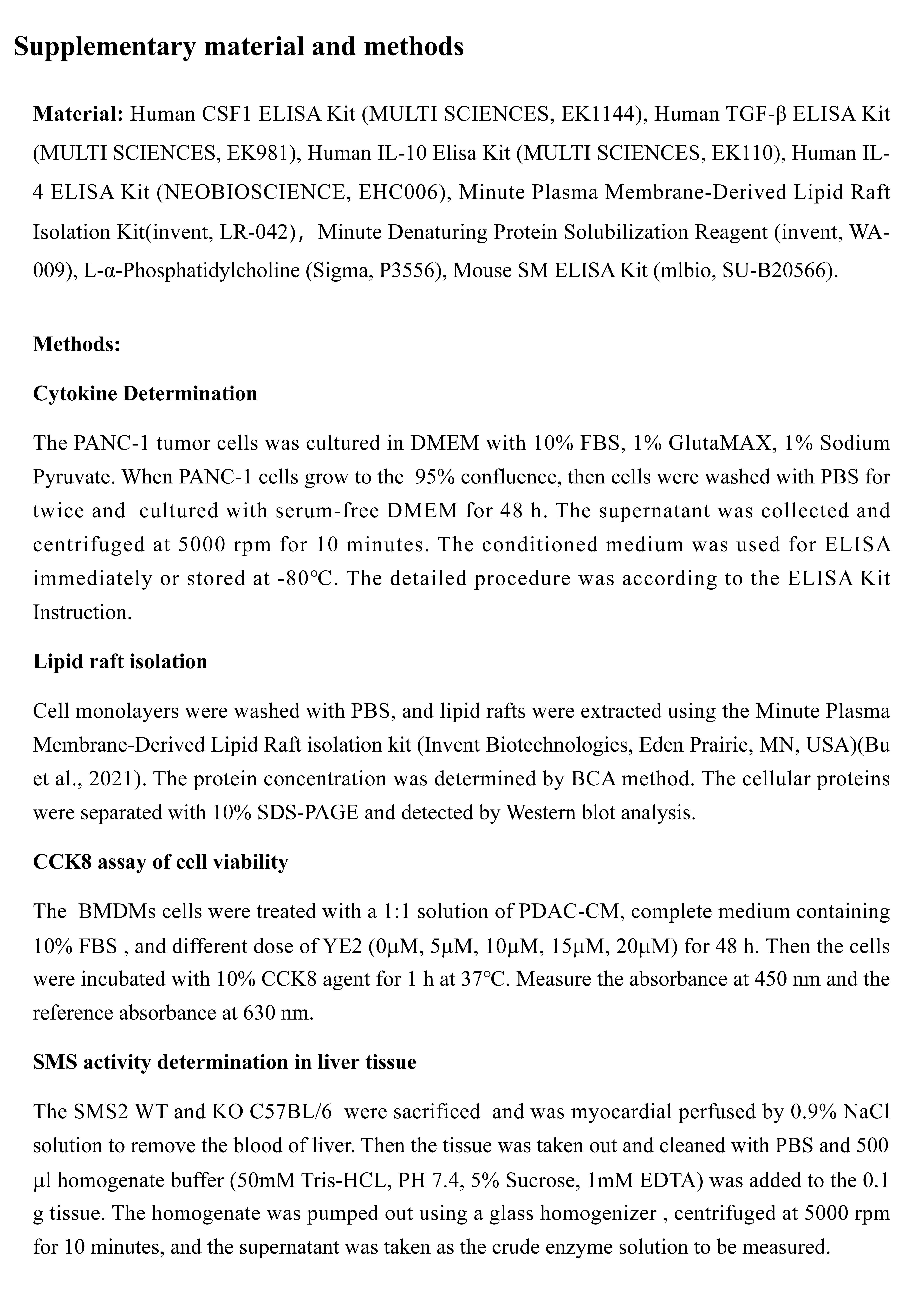

Supplement: Supplementary file 2 [file Image1.JPEG]

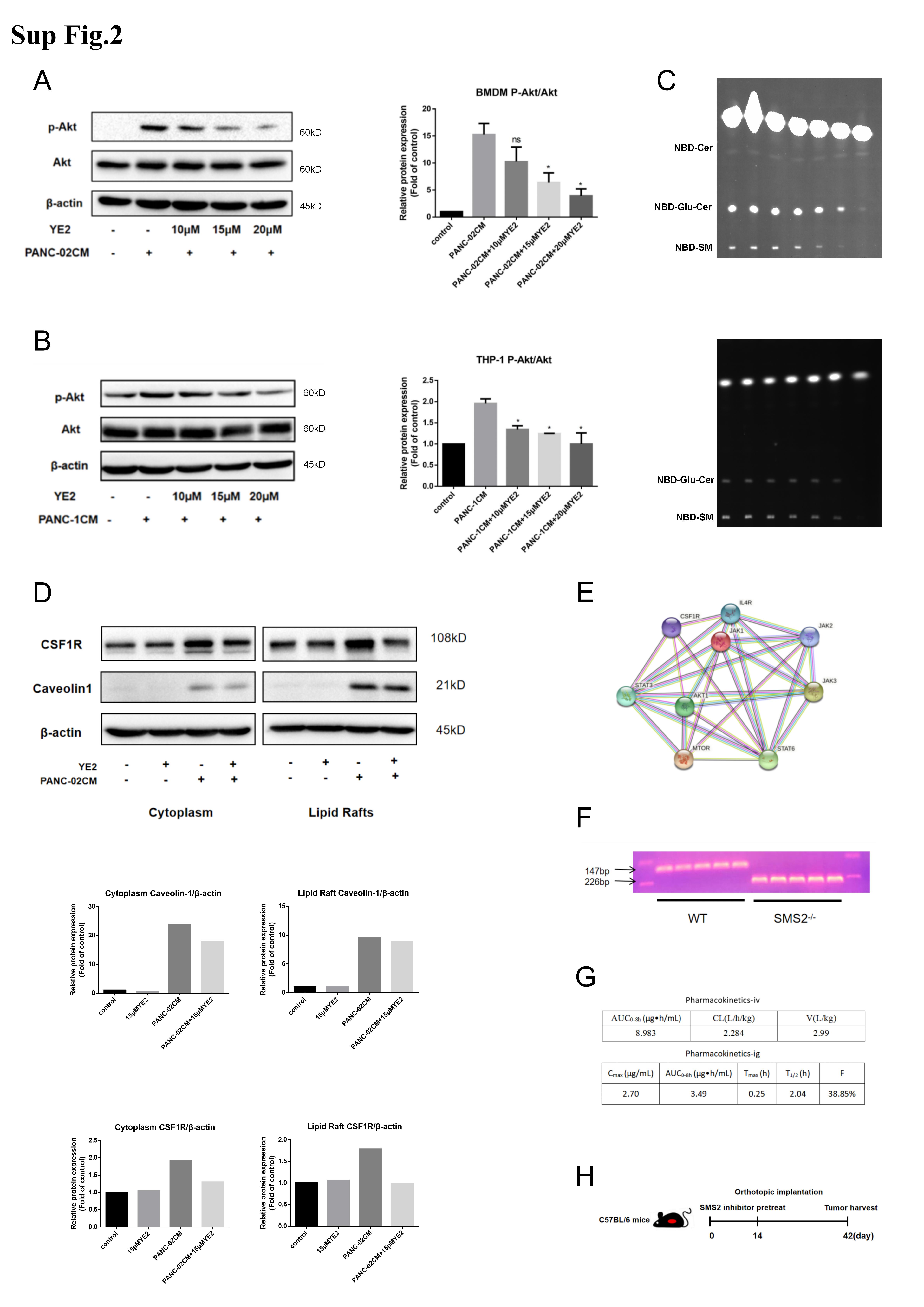

Supplement: Supplementary file 3 [file Image4.JPEG]

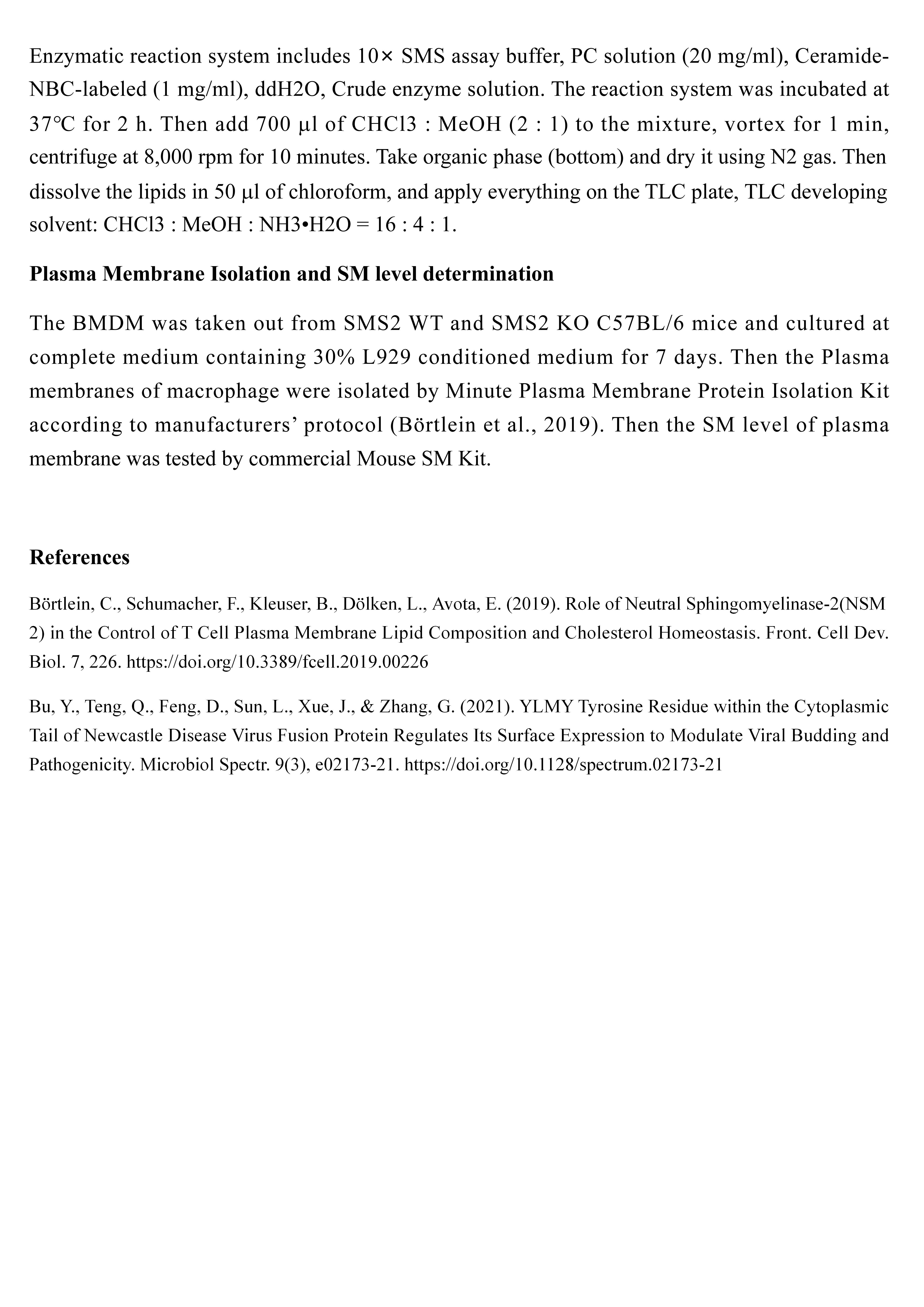

Supplement: Supplementary file 4 [file Image2.JPEG]

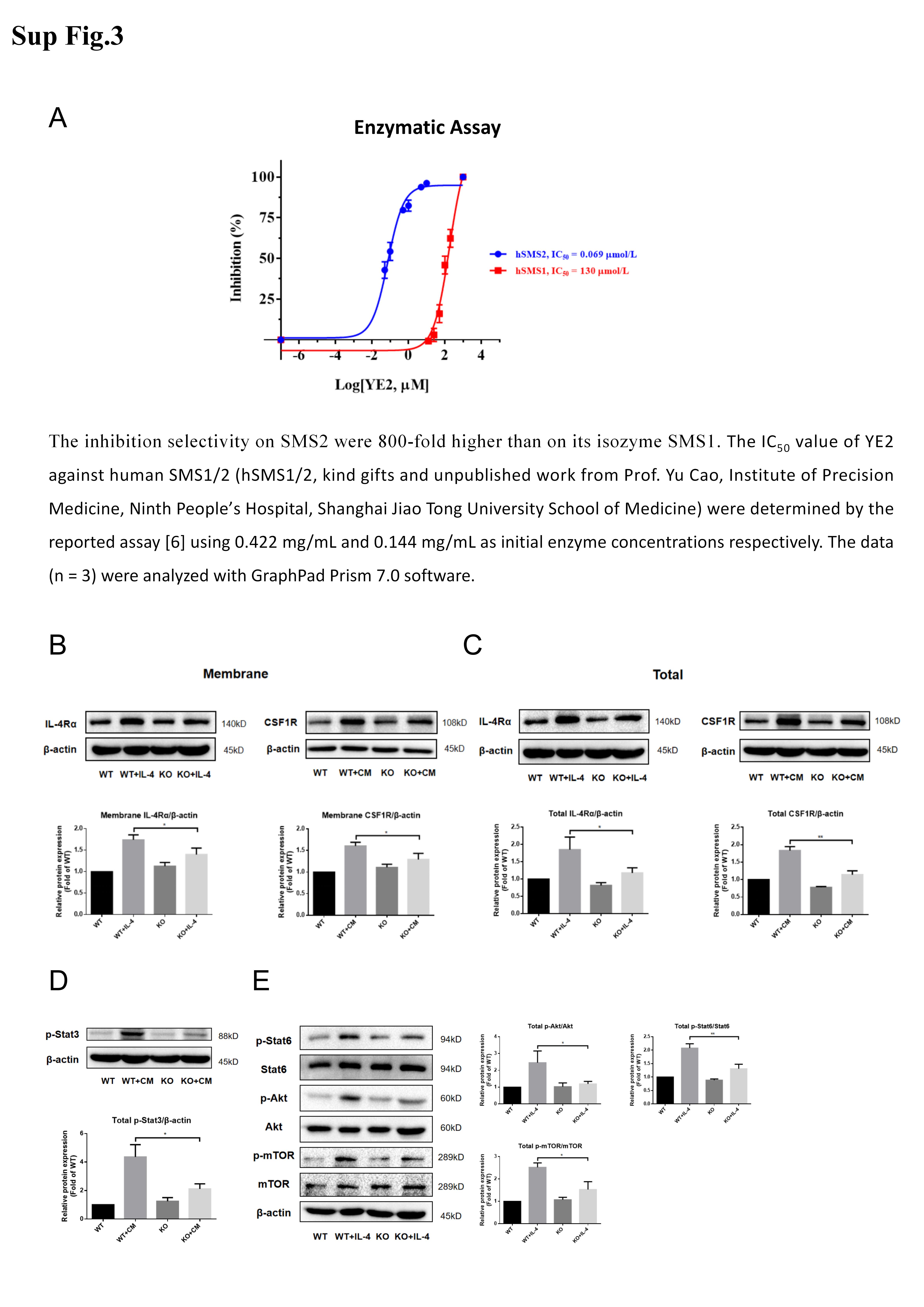

Supplement: Supplementary file 5 [file Image5.JPEG]

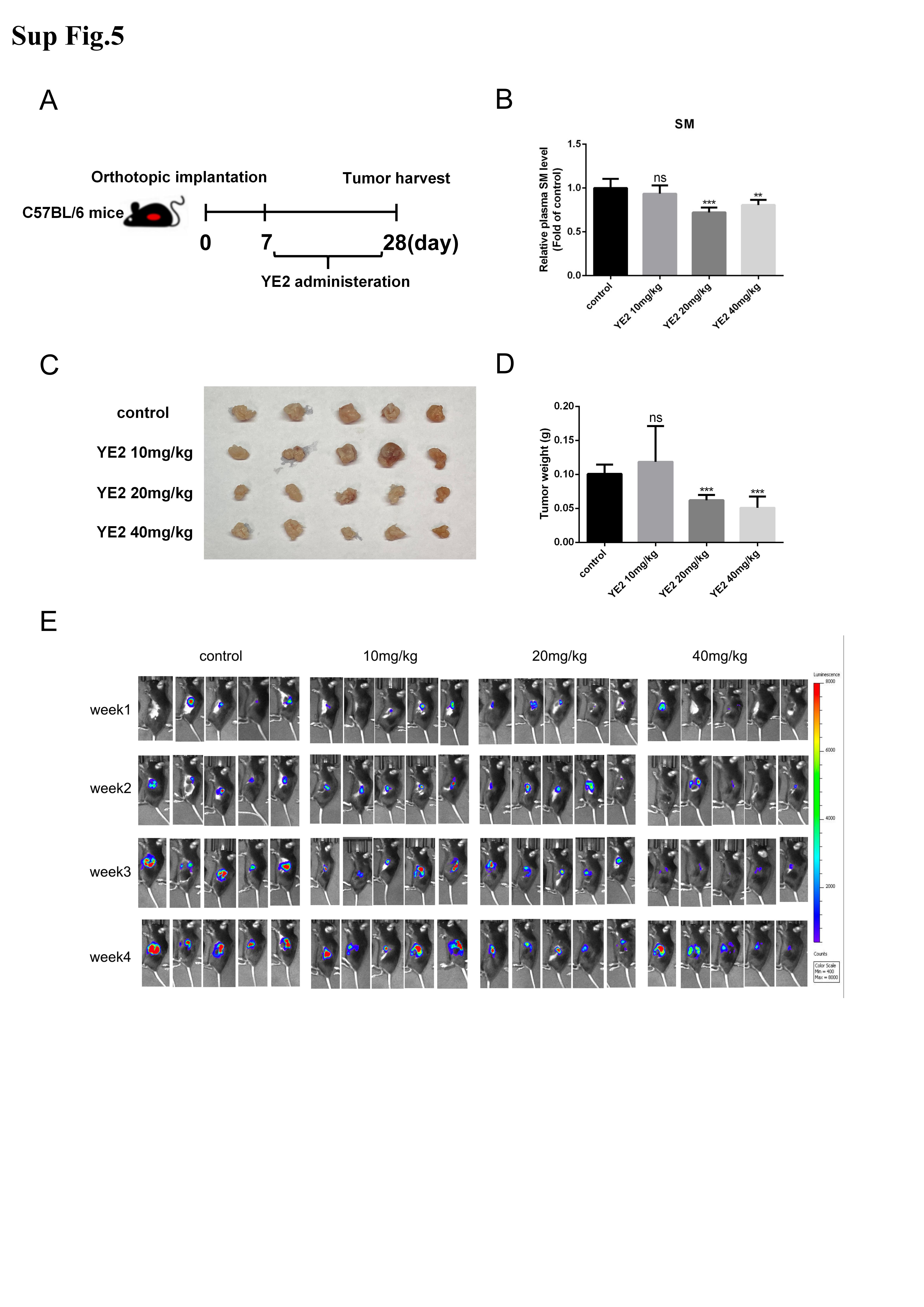

Supplement: Supplementary file 6 [file Image7.jpg]

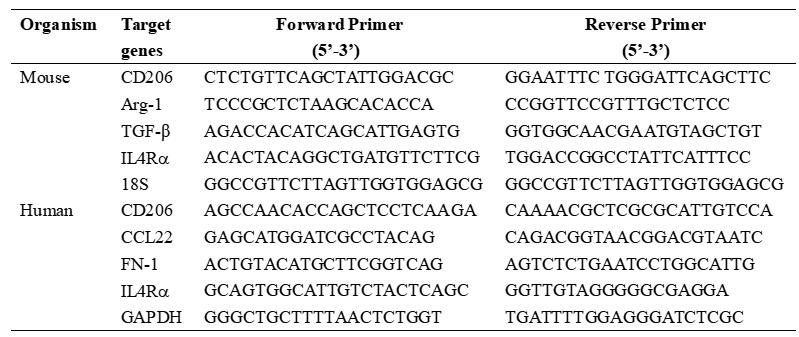

Supplement: Supplementary file 7 [file Image8.JPEG]

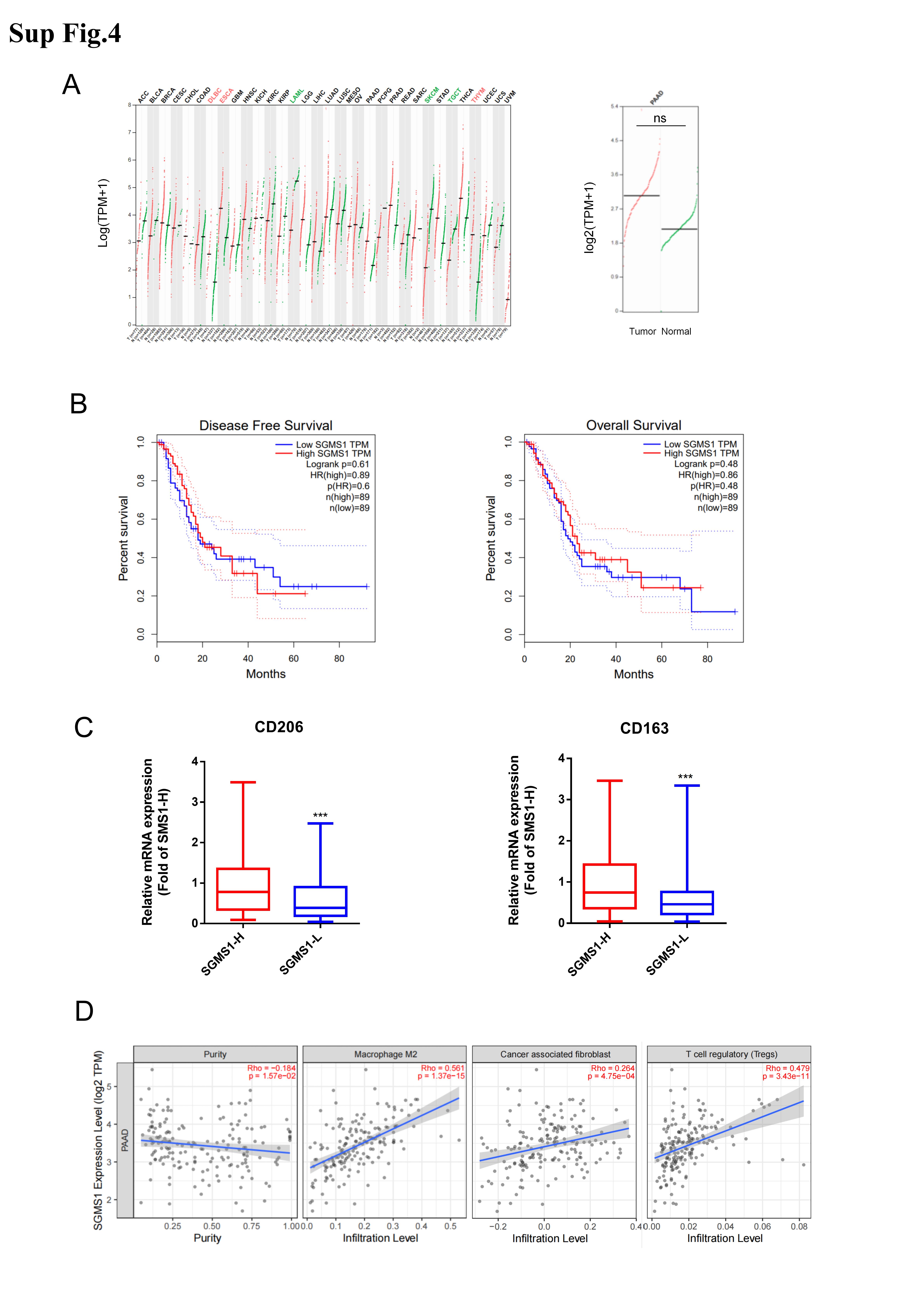

Supplement: Supplementary file 8 [file Image6.JPEG]
